# Prophylactic oophorectomy is recommended in female patients with pseudomyxoma peritonei of an appendiceal origin

**DOI:** 10.3389/fonc.2025.1485694

**Published:** 2025-07-24

**Authors:** Fengxian Fu, Xulan Ma, Zhen Weng, Yiyan Lu, Hongbin Xu, Ruiqing Ma

**Affiliations:** ^1^ Department of Gynecology, Aerospace Center Hospital, Beijing, China; ^2^ Ministry of Education (MOE) Engineering Center of Hematological Disease, Soochow University, Suzhou, China; ^3^ Cyrus Tang Hematology Center, Soochow University, Suzhou, China; ^4^ Collaborative Innovation Center of Hematology, Soochow University, Suzhou, China; ^5^ National Clinical Research Center for Hematologic Diseases, The First Affiliated Hospital of Soochow University, Suzhou, China; ^6^ Department of Pathology, Aerospace Center Hospital, Beijing, China; ^7^ Department of Myxoma, Aerospace Center Hospital, Beijing, China

**Keywords:** pseudomyxoma peritonei, cytoreductive surgery plus hyperthermic intraperitoneal chemotherapy, prognosis, hysterectomy and bilateral salpingooophorectomy, macroscopic and microscopic involvement

## Abstract

**Objective:**

To analyze the clinical efficacy of hysterectomy and bilateral salpingo-oophorectomy (BSO) in female patients with pseudomyxoma peritonei (PMP) of an appendiceal origin as well as to identify the prognostic factors associated with overall survival (OS) to reduce adverse physical or psychological effects on women.

**Methods:**

A retrospective analysis was conducted to evaluate the prognostic factors in female patients with PMP of an appendiceal origin who underwent cytoreductive surgery (CRS) and hyperthermic intraperitoneal chemotherapy (HIPEC) including BSO at our hospital between January 2011 and December 2020. Patients were also divided into high and low grade group according to pathology subtype. The data were analyzed by using Chi-square, univariate and multivariate cox regression methods, and survival was evaluated by Kaplan-Meier method.

**Results:**

We included 178 patients with PMP of an appendiceal origin who underwent BSO, and 134 (75.3%) had histopathological confirmed ovarian metastases. In the low-grade group, ovarian involvement and completeness of cytoreduction (CCR) acted as independent predictors of poor prognosis for OS. In normal appearing uterus, ovaries, fallopian tubes, pathologic disease was still found in 15.4%, 7.8%, and 22.8% respectively. In abnormal appearing uterus, ovaries, fallopian tubes, pathologic disease was found in 95.5%, 96.6%, and 94.2% respectively. Moreover, multivariate analysis revealed that a high-grade histopathological subtype of PMP and CCR 2/3 were independent predictors of OS.

**Conclusion:**

Based on these data, strong consideration should be given to removal of gynecologic organs in cases of appendiceal peritoneal disease even when normal in appearance, after taking into account each individual patient’s fertility concerns.

## 1 Introduction

Pseudomyxoma peritonei (PMP) is a rare condition with an estimated incidence of 1–2 cases per million per year and is characterized by the extensive growth of mucinous tumors in the peritoneal cavity, whereas the appendix is the most common primary site for PMP (1, 2). In clinical practice, appendiceal mucinous neoplasms derived PMP is often misdiagnosed as primary ovarian tumors (3, 4). With the revised diagnostic criteria, the prevalence of mucinous ovarian cancer (MOC) has decreased from <10% to 3–5% of all epithelial ovarian cancers (5). It is reported that over 80% of MOCs are metastatic and the most common primary sites being the lower gastrointestinal tract, appendix, stomach, and pancreas (6, 7). The ovary undergoes monthly ovulation that provides a favorable environment for tumor cell implantation (8). Therefore, bilateral salpingo-oophorectomy (BSO) is an essential modality of complete cytoreductive surgery (CRS) in most cases (9, 10). However, a significant proportion of female patients with PMP are of childbearing age, and routine BSO could result in infertility and affect cardiovascular health (11). It is therefore important to analyze the prognostic impact of ovarian metastases and BSO in these patients. Therefore, here we analyzed the clinicopathological characteristics of PMP so as to provide guidance for female patients and their providers.

## 2 Materials and methods

### 2.1 Ethical approval

This study protocol was approved by the Ethics committee of the Aerospace Center Hospital, Beijing, China (No. 20190301-YN-09). Written informed consent was obtained from all patients with regards to using their clinical data and images.

### 2.2 Patient selection

We conducted a retrospective analysis of evaluate the prognostic factors in female patients with PMP of an appendiceal origin who underwent CRS and hyperthermic intraperitoneal chemotherapy (HIPEC) including BSO at our hospital between January 2011 and December 2020. For diagnosis purpose, two experienced pathologists confirmed the histological origin of the ovarian mucinous tumor of female patients with appendiceal mucinous neoplasm-derived PMP who underwent CRS and HIPEC according to the pathological classification by Carr et al. (12).

The exclusion criteria were as follows (1): non-appendiceal origin PMP; (2) a history of oophorectomy or hysterectomy before CRS; (3) unilateral/bilateral ovaries are preserved during CRS; (4) incomplete follow-up records; (5) the presence of other severe organic diseases, including heart, brain, liver and kidney.

### 2.3 Surgical procedure

All CRS+HIPEC procedures were conducted by a designated team of 4 surgeons with expertise in peritoneal carcinomatosis treatment and at least 1 gynecologic oncologist. After inducing general anesthesia, a midline incision was made from the xiphoid to the pubic symphysis, followed by an exploration of the disseminated content of peritoneal metastases from the diaphragmatic peritoneum to the pelvic peritoneum, including the nature and amount of ascites as well as the location and size of the tumor. Based on these properties of the content, the peritoneal cancer index (PCI) was evaluated (13). Subsequently, maximal CRS was conducted for the curative or palliative resection of the primary tumor with acceptable margins, any involved adjacent structures, lymphadenectomy, and peritonectomy (**Supplementary Figure 1**). BSO was performed in all the patients except those with normal appearance of fallopian tubes or ovaries or normal intraoperative frozen pathology or require to preserve during the surgery. If the uterus appeared normal and did not show adherence to the surrounding tissues, and if the patient was desirous to preserve it, the organ was retained. After CRS, completeness of cytoreduction (CCR) was evaluated based on the size of the residual tumor (14). HIPEC was then implemented with two inflow and two outflow catheters placed in the peritoneal cavity and connected to the machine (Jilin Minda Company, RHL-2000B, Changchun, Jilin, China). Mitomycin (20 mg/m^2^) was warmed to 41-42°C and circulated intraperitoneally for 60–90 min after a temporary approximation of the abdominal skin (15). Next, the reconstruction of the digestive tract or urinary tract and the intestinal stoma was performed, followed by the placement of abdominal drainage tubes and suturing of the incision.

### 2.4 Adjuvant therapy

Patients with high-grade PMP were treated by initiating adjuvant intravenous chemotherapy 4 weeks later. The following XELOX regimen was preferred: oxaliplatin 130 mg/m^2^ accompanied by oral Xeloda (1000 mg/m^2^) for 14 days, followed by a break period of 1 week. Postoperative intravenous chemotherapy was not recommended in patients with low-grade PMP (16).

### 2.5 Follow-up protocol

The frequency of follow-up was twice a year in the first 5 years of surgery and then once a year thereafter. The frequency of follow-up was kept the same for patients with and without ovarian metastases. The follow-up monitoring included an examination of the general status, tumor response, quality of life, and survival data. The last follow-up was conducted in December 2022 via telephone or at an outpatient clinic. The follow-up rate was 100%.

### 2.6 Study parameters

The clinicopathological parameters for the analysis included a collection of demographic details, clinical history, the details of previous systemic chemotherapy (PSC), prior surgical score (PSS) (17), intraoperative PCI (13), CCR (14), the details of HIPEC, the histopathological subtype of PMP, macroscopic and microscopic involvement of the uterus, fallopian tubes or ovaries, overall survival (OS), and the follow-up duration. Pathological diagnosis was made in accordance with the 2016 Peritoneal Surface Oncology Group International criteria (12), as acellular mucin, patients with acellular mucin or low-grade mucinous carcinoma peritonei were classified as having low-grade disease; patients with high-grade mucinous carcinoma peritonei without and with signet ring cells were classified as having high-grade disease. OS was calculated from the date of surgery until the time of death or the last follow-up.

### 2.7 Statistical analyses

Statistical analyses were performed using the SPSS version 24.0 (IBM Corporation, Armonk, NY, USA). Continuous data were presented as the median and range and compared by the independent-sample *t*-test or Mann–Whitney U-test according to whether the data was normally distributed. Categorical variables were expressed as number and percentages and were compared by Chi-squared (*χ^2^*) or Fisher’s exact test, as deemed appropriate. Survival was evaluated by the Kaplan-Meier method and compared by the log-rank tests between group. Cox proportional hazards model was applied to perform multivariate analyses with stepwise forward selection, and those factors with a p-value <0.1 in univariate analysis were further included in multivariate analyses. Subgroup analysis was also performed based on pathological type. *P* < 0.05 was considered to indicate statistical significance.

## 3 Results

### 3.1 Clinicopathological characteristics

**Figure 1:**
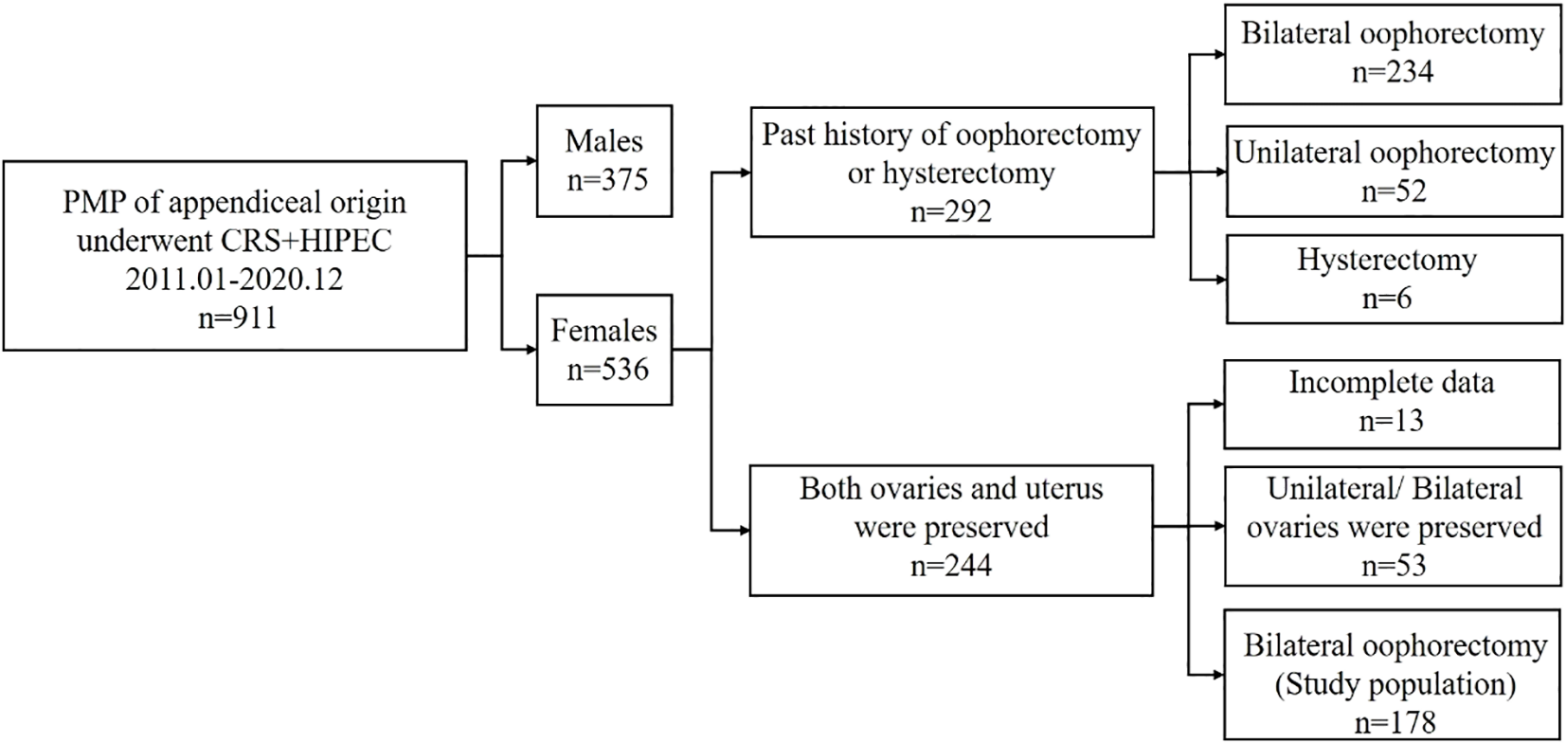
Flow chart depicting the screening process of the study population.

During the period of present study, a total of 911 patients with PMP of an appendiceal origin underwent CRS + HIPEC, of which 58.8% were women. Of these patients, 234 (43.7%), 52 (9.7%), and 6 (1.1%) had a previous history of bilateral, unilateral oophorectomy, and hysterectomy, respectively, whereas 244 (45.5%) patients had both ovaries and a uterus before the surgery. Of the 244 patients with preserved ovaries and a uterus prior to CRS-HIPEC, 53 patients were excluded for unilateral and bilateral ovaries preservation. After removal of 13 patients with incomplete data finally, and after applying the selection criteria, 178 patients were included in present study (**Figure 1**).

The demographic and clinical characteristics of the patients are presented in **Table 1**. The median age of the patients was 61 years (age range: 27–84 years). Of the patients, 86% were postmenopausal. The median BMI was 23.51 kg/m^2^ (range: 18-36). The median time from diagnosis to surgery was 3 months, with a range of 0–138 months. Prior to CRS and HIPEC, 21.3% of the patients underwent preoperative systemic chemotherapy (PSC), and most of the patients (86%) had a PSS score of 0, 1, or 2, while 14% had a score of 3. Moreover, of these patients, 37.1% had a PCI score of ≤20, while 62.9% had a score of >20. The CCR score was (0 or 1) and (2 or 3) in 49.4% and 50.6% of the patients, respectively. A total of 36% of the patients did not undergo a hysterectomy. The low and high grade histopathological subtype PMP was respectively present in 62.4% and 37.6% of patients. The negative, low and high grade histopathologic subtype of the uterus was respectively present in 14.6%, 32.6% and 16.9% of patients except 36% of censored case. The negative, low and high grade histopathological subtype of the ovary was respectively found in 24.7%, 47.8% and 27.5% of patients. The negative, low and high grade histopathological subtype of the fallopian tube was respectively observed in 28.7%, 48.3% and 23% of patients.

**Table 1:** Patient demographic data.

Characteristics	No. of Patients (n = 178)
Status	
Survival	91 (51.1%)
Death	87 (48.9%)
OS (months)	
median (range, months)	41 (0–116)
OS rate	
2-year	145 (81.5%)
3-year	116 (65.2%)
5-year	45 (25.3%)
Age (years)	
Median (range, years old)	61 (27–84)
<60	72 (40.4%)
≥60	106 (59.6%)
Menstrual status	
premenopausal	25 (14%)
postmenopausal	153 (86%)
BMI (kg/m^2^)	
Median (range)	23.51 (18–36)
<28	146 (82%)
≥28	27 (15.2%)
censored	5 (2.8%)
Duration of symptoms (months)	
Median (range)	3 (0–138)
<12	127 (71.3%)
≥12	51 (28.7%)
PSC	
Yes	38 (21.3%)
PSS	
0/1/2	153 (86%)
3	25 (14%)
PCI (months)	
median (range)	24.5 (0–39)
≤20	66 (37.1%)
>20	112 (62.9%)
CCR	
0/1	88 (49.4%)
2/3	90 (50.6%)
Hysterectomy	
Yes	114 (64%)
Histopathological subtype of PMP	
Low-grade	111 (62.4%)
High-grade	67 (37.6%)
Histopathological subtype of the uterus	
Negative	26 (14.6%)
Low-grade	58 (32.6%)
High-grade	30 (16.9%)
NA	64 (36%)
Histopathological subtype of the ovary	
Negative	44 (24.7%)
Low-grade	85 (47.8%)
High-grade	49 (27.5%)
Histopathological subtype of the fallopian tube	
Negative	51 (28.7%)
Low-grade	86 (48.3%)
High-grade	41 (23%)

OS, overall survival; BMI, body mass index; PSC, previous systemic chemotherapy; PSS, prior surgical score; PCI, peritoneal cancer index; CCR, completeness of cytoreduction; PMP, pseudomyxoma peritonei; NA, not available.

### 3.2 Survival outcomes

The median OS for the patients was 41 months (range: 0–116 months). Furthermore, the 2-, 3-, and 5-year survival rates were 81.5%, 65.2%, and 25.3%, respectively. Moreover, we also listed the demographic data about 51 patients without ovarian involvement. An increased 2-, 3-, and 5-year survival rates could be found in this group of patients.

### 3.3 Comparisons of the uterus, fallopian tubes and ovaries involvement of in PMP patients with different histopathological subtypes

Comparisons of microscopic involvement of the uterus, fallopian tubes and ovaries in patients with low-grade and high-grade histopathologic subtypes of PMP are presented in **Table 2**. The results indicated significant differences between the 2 groups on microscopic ovarian involvement (Yes: 69.4% vs. 85.1%, *p* = 0.019) and the histopathologic subtype of ovarian involvement (high-grade: 1.3% vs 84.2%, *p* < 0.001), but not for uterus or fallopian tubes involvement.

**Table 2:** Comparison of uterine and adnexal involvements of different histopathological subtypes in patients with PMP.

Variable	Low-grade (n = 111)	High-grade (n = 67)	χ^2^	*p-*value
Uterus involvement				
Macroscopic disease			0.337	0.561
Yes	53 (47.7)	35 (52.2)		
Microscopic disease			0.106	0.744
No	16 (14.4)	10 (14.9)		
Yes	51 (45.9)	37 (55.2)		
NA	44 (39.6)	20 (29.9)		
Ovarian involvement				
Macroscopic disease			3.162	0.075
Yes	74 (66.7)	53 (79.1)		
Bilateral	68 (91.9)	48 (90.6)	0.069	1.000
Unilateral	6 (8.1)	5 (9.4)	0.782	0.782
Microscopic disease			5.538	0.019*
Yes	77 (69.4)	57 (85.1)		
Low-grade	76 (98.7)	9 (15.8)	97.071	<0.001*
High-grade	1 (1.3)	48 (84.2)		
Bilateral	70 (90.9)	48 (84.2)	1.398	0.237
Unilateral	7 (9.1)	9 (15.8)	0.042	1.000
Fallopian tube involvement				
Macroscopic disease			0.233	0.629
Yes	74 (66.7)	47 (70.1)		
Microscopic disease			0.565	0.452
Yes	77 (69.4)	50 (74.6)		

PMP, pseudomyxoma peritonei; BMI, body mass index; PSC, previous systemic chemotherapy; PSS, prior surgical score; PCI, peritoneal cancer index; CCR, completeness of cytoreduction; NA, not available; *p<0.05.

(n = 178).

### 3.4 Comparison of baseline characteristics of patients with and without pathologic confirmed ovarian involvement

The baseline characteristics of patients with (n = 134, 75.3%) and without (n = 44, 24.7%) pathologic confirmed ovarian involvement were also compared (**Table 3**). Ovarian involvement was correlated with the survival statue (alive:70.5% vs. 44.8%, *p* = 0.003), moreover, several other factors showed significant differences between the 2 groups, including PCI (>20: 20.5% vs. 76.9%, *p* < 0.001), CCR (2/3:13.6% vs. 62.7%, *p* < 0.001), histopathologic subtype of PMP (high-grade:22.7 vs.42.5%, *p* = 0.019), uterus involvement by macroscopic (Yes:4.5% vs 64.2%, *p* < 0.001) or microscopic disease (Yes:4.5% vs 64.2%, *p* < 0.001), and fallopian tube involvement by macroscopic (Yes:6.8% vs. 88.1%, *p* < 0.001) or microscopic disease (Yes: 11.4% vs. 91%, *p* < 0.001). Interestingly, among those patients with ovarian involvement, 77 out of 134 are low-grade PMP. However, the ratio of ovarian involvement was higher in high-grade PMP than that in low-grade PMP [57/(57 + 10)=85% vs 77/(77 + 34)=69%].

**Table 3:** Comparison of the baseline characteristics between PMP patients with and without pathologic confirmed ovary involvement.

Variable	Non-involvement (n = 44)	Involvement (n = 134)	χ^2^	*p-*value
Status			8.741	0.003*
Survival	31 (70.5)	60 (44.8)		
Death	13 (29.5)	74 (55.2)		
Age (years)			2.213	0.137
<60	22 (50)	50 (37.3)		
≥60	22 (50)	84 (62.7)		
BMI (kg/m^2^)			0.119	0.730
<28	37 (84.1)	109 (81.3)		
≥28	6 (13.6)	21 (15.7)		
NA	1 (2.3)	4 (3)		
Duration of symptoms (months)			3.134	0.077
<12	36 (81.8)	91 (67.9)		
≥12	8 (18.2)	43 (32.1)		
PSC			2.070	0.150
Yes	6 (13.6)	32 (23.9)		
PSS			0.008	0.928
0/1/2	38 (86.4)	115 (85.8)		
3	6 (13.6)	19 (14.2)		
PCI			45.180	<0.001*
≤20	35 (79.5)	31 (23.1)		
>20	9 (20.5)	103 (76.9)		
CCR			31.881	<0.001*
0/1	38 (86.4)	50 (37.3)		
2/3	6 (13.6)	84 (62.7)		
Histopathologic subtype of PMP			5.538	0.019*
Low-grade	34 (77.3)	77 (57.5)		
High-grade	10 (22.7)	57 (42.5)		
Uterus involvement				
Macroscopic disease			47.123	<0.001*
Yes	2 (4.5)	86 (64.2)		
Microscopic disease			35.875	<0.001*
No	12 (27.3)	14 (10.4)		
Yes	2 (4.5)	86 (64.2)		
NA	30 (68.2)	34 (25.4)		
Fallopian tube involvement				
Macroscopic disease			100.432	<0.001*
Yes	3 (6.8)	118 (88.1)		
Microscopic disease			102.876	<0.001*
Yes	5 (11.4)	122 (91)		

PMP, pseudomyxoma peritonei; BMI, body mass index; PSC, previous systemic chemotherapy; PSS, prior surgical score; PCI, peritoneal cancer index; CCR, completeness of cytoreduction; NA, not available; *p<0.05.

(n = 178).

### 3.5 Relationship between macroscopic and microscopic appearance on involvement of uterus, fallopian tubes and ovaries

The corresponding relationship between the macroscopic and microscopic appearance on involvement of the uterus, fallopian tubes and ovaries is depicted in **Table 4**. In general, among the patients with normal uterine appearance, 28.9% underwent a hysterectomy, of which 15.4% patients had pathologic confirmed uterine involvement. In cases with an abnormally appearing uterus,95.5% of them were confirmed to have disease involvement. When the appearance of the bilateral ovaries was normal, 15.7% of ovaries had evidence of disease, of which 7.8% had the involvement of both ovaries, 5.9% had right ovary involvement, and 2% had left ovary involvement. When the appearance of bilateral ovaries was abnormal, 96.6% had bilateral ovarian involvement, only 0.9% had the unilateral left ovarian involvement and 2.6% had right ovarian involvement. When the unilateral ovary appeared abnormal (Left:4%, Right: 4.7%, total=8.7%), 18.2%(2/11) of the patients had the contralateral ovary involved (**Supplementary Table 2**). When the appearance of bilateral fallopian tubes was normal, 22.8% were involved, and in case of abnormal appearance, 94.2% were involved.

**Table 4:** The relationship between macroscopic and microscopic involvement of the uterus, ovaries and fallopian tubes.

Organ	No macroscopic disease			Macroscopic disease		
Uterus	90			88		
Surgical resection	26 (28.9%)			88 (100%)		
Microscopic disease	No	Yes		No	Yes	
	22 (84.6%)	4 (15.4%)		4 (4.5%)	84 (95.5%)	
Histopathological subtype	Negative	Low-grade	High-grade	Negative	Low-grade	High-grade
	22 (84.6%)	1 (3.9%)	3 (11.5%)	4 (4.5%)	57 (64.8%)	27 (30.7%)
Ovary	51			127		
Surgical resection	51 (100%)			127 (100%)		
Microscopic disease	No	Yes		No	Yes	
	43 (84.3%)	8 (15.7%)		1 (0.8%)	126 (99.2%)	
Histopathological subtype	Negative	Low-grade	High-grade	Negative	Low-grade	High-grade
	43 (84.3%)	4 (5.9%)	4 (5.9%)	1 (0.8%)	81 (63.8%)	45 (35.4%)
Fallopian tube	57			121		
Surgical resection	57 (100%)			121 (100%)		
Microscopic disease	No	Yes		No	Yes	
	44 (77.2%)	13 (22.8%)		7 (5.8%)	114 (94.2%)	
Histopathological subtype	Negative	Low-grade	High-grade	Negative	Low-grade	High-grade
	44 (77.2%)	8 (14%)	5 (8.8%)	7 (5.8%)	78 (64.4%)	36 (29.8%)

% represents percentages of total number of parameters at previous level.

(n = 178).

### 3.6 Univariate analysis of OS

**Figure 2:**
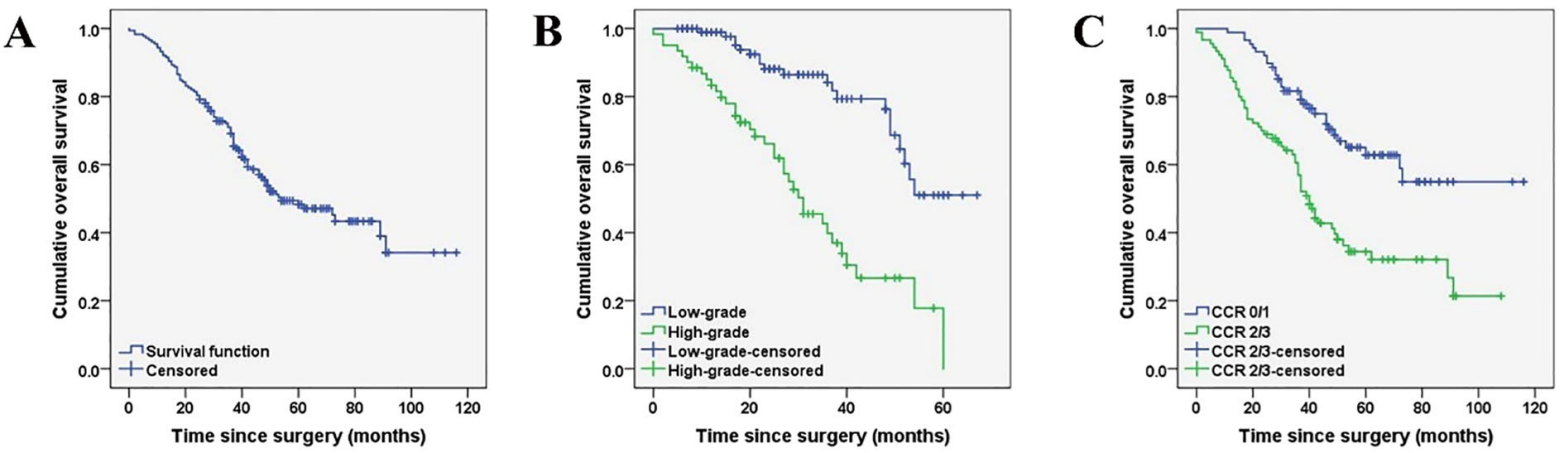
Kaplan-Meier curves depicting the overall survival **(A)** of female patients with PMP of an appendiceal origin and comparison of the overall survival based on histopathological subtypes of PMP (low-grade versus high-grade) (B*), CCR type (0/1 versus 2/3) (C*). (*P < 0.05).

**Figure 3:**
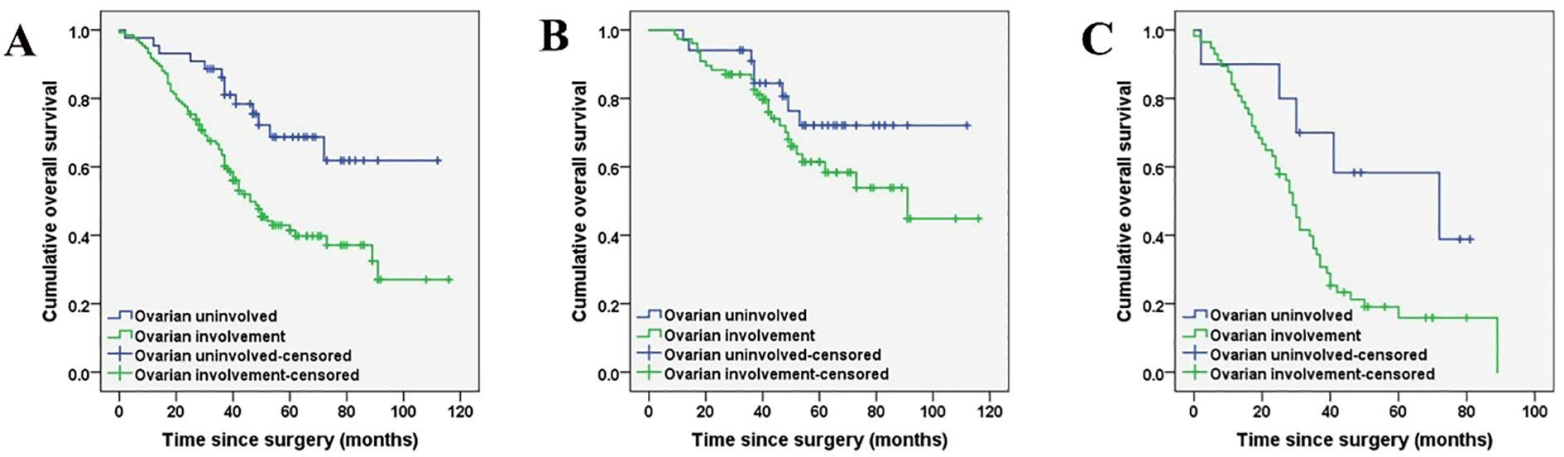
Kaplan-Meier curves indicating ovarian involvement in the whole cohort (n = 178) **(A)**, low-grade PMP group (n = 111) (B*), and high-grade PMP group (n = 67) **(C)**. (*P < 0.05).

The prognostic factors affecting the OS of the entire cohort identified by univariate analysis are shown in **Table 5**. Among all the patients included in the study, the histopathological subtype of PMP (HR: 4.231, 95%CI:2.730-6.556, *p* < 0.001), pathologic confirmed ovarian involvement (HR: 2.401, 95%CI: 1.330-4.335, *p* = 0.003), PCI (HR: 2.321, 95%CI: 1.429-3.770, *p* < 0.001), and CCR (HR: 2.478, 95%CI: 1.590-3.863, *p <*0.001) were all found to be significant prognostic factors for OS (**Figures 2**, **3**). Subgroup univariate analysis of OS was also performed based on high (n = 67) and low-grade (n = 111) subtype of PMP. The prognostic factors statistically significant for OS differed between these two groups. In the low-grade group, pathologic confirmed ovarian involvement (HR: 0.206, 95%CI: 0.055–0.765, *p* = 0.018) and CCR (HR: 8.064, 95%CI: 1.980–32.837, *p*=0.004) showed significance, whereas in the high-grade group, ovarian involvement (HR: 2.368, 95%CI: 0.892–6.284, *p* = 0.042) was the only significant factor.

**Table 5:** Univariate and multivariate analyses of factors affecting the OS.

Variable	Univariate HR (95% CI)	Univariate *p-*value	Multivariate HR (95% CI)	*p-*value
Histopathological subtype of PMP				
Low-grade	reference		reference	
high-grade	4.231 (2.730-6.556)	<0.001	3.819 (2.458–5.933)	<0.001*
Pathologic confirmed ovarian involvement				
no	reference			
Yes	2.401 (1.330-4.335)	0.004		
PCI				
≤20	reference			
>20	2.321 (1.429-3.770)	0.001		
CCR				
0/1	reference		reference	
2/3	2.478 (1.590-3.863)	<0.001	2.091 (1.337–3.271)	0.001*
Hysterectomy				
no	reference			
Yes	1.195 (0.765-1.865)	0.434		

OS, overall survival; HR, hazard ratio; CI, confident interval; PMP, pseudomyxoma peritonei; PCI, peritoneal cancer; CCR, completeness of cytoreduction.

(n = 178).

### 3.7 Multivariate analysis of OS

A Cox regression model was utilized for multivariate analysis of the entire cohort, which revealed that the high-grade histopathologic subtype of PMP (HR:3.819, 95%CI: 2.458–5.933, *p* < 0.001) and CCR score of 2/3 (HR: 2.091, 95%CI: 1.337–3.271, *p* < 0.001) were independent predictors of poor prognosis for OS. Moreover, subgroup multivariate analysis confirmed that in the low-grade group, pathologic confirmed ovarian involvement (HR: 0.243, 95%CI: 0.068–0.867, *p* = 0.029) and CCR (HR: 13.943, 95%CI: 3.891–49.970, *p* < 0.001) were both independent predictors of poor prognosis for OS.

## 4 Discussion

The recommended treatment for PMP is combining CRS with HIPEC (10, 18). However, it is unclear whether routine BSO should be performed during CRS and HIPEC for PMP in women, especially for those who are premenopausal and with normal-looking ovaries. In addition to fertility, BSO can also have psychological and physical impacts. As previously mentioned (19, 20), only a very small proportion of the female reproductive system involved with PMP originated from the primary ovary, and most are secondary ovarian metastases of appendicular origin. Therefore, here we focus on the long-term influence of appendicular-derived PMP on the female reproductive system, which was rarely studied.

High-grade histopathologic subtypes of PMP and CCR 2/3 was found as independent OS predictors, which are consistent with our previous reports (21). Moreover, we observed a relationship between ovarian involvement and survival status. A retrospective study with 71 female patients who were appendiceal mucinous neoplasm reported that PCI and serum lactate dehydrogenase (LDH) levels are prognosis predictors, while ovarian involvement does not significantly affect prognosis. Similarly, Bignell et al. investigated the survival of patients with ovarian metastases from low-grade appendix tumors or colorectal cancer treated with CRS and HIPEC and reported similar survival rates compared to those without ovarian metastases (22). These findings support our results about BSO might not be crucial for the prognosis of female PMP patients with an appendiceal origin (23). Furthermore, whether the pathologic confirmed ovarian involvement had the same prediction efficacy as the pathological pattern of PMP is also unknown. We found here that high-grade PMP patients were more likely to have microscopic ovarian involvement and a higher ovarian pathological grade. Mehta et al. discovered that occult malignancy was present in 17% of cases bilateral normal appeared ovaries and in 45% of normal appeared ovaries when contralateral ovary involved (24). We found here that 28.7% bilateral ovarian involved cases had normal appearance, among which 15.7% had occult malignancies. In contrast, among 71.3% of abnormal appeared ovarian PMP patients, only 0.8% did not have ovarian involvement. Pathological involvement was observed in all the patients with bilateral abnormal appeared ovarian. When the macroscopic abnormality was identified in unilateral ovary, pathologic involvement could be found in 90.9% of local ovary and 18.2% of the contralateral ovary. These results suggest that a risk of pathologic ovarian involvement even when the bilateral ovaries have normal appearance. Moreover, a significant likelihood of pathological involvement in corresponding and contralateral ovary in cases with the unilateral abnormal appeared ovary. Therefore, bilateral oophorectomy is recommended. A study involving 33 female PMP patients (aged <41 years) treated CRS and HIPEC (8) reported a 43% ovarian preservation for future pregnancy desire, resulting in one successful birth, suggested that ovarian preservation may be an option for low-grade PMP patients for future pregnancy desire. However, another study revealed that when peritoneal carcinomatosis of colorectal or appendiceal origin was confirmed, at least 52% of ovaries would have synchronous metastases, resulting in a significantly lower disease-free survival. Therefore, oophorectomy was strongly recommended (9). Our subgroup analysis indicated that ovarian involvement could predict the survival in the low-grade PMP and affected the high-grade survival, implying its prognosis impact. Based on these findings, we proposed a comprehensive consideration of oophorectomy.

In PMP patients undergoing CRS, of 178 patients, 50.6% with normal appeared uterus and 26 patients underwent hysterectomy due to adhesion of the adjacent organs or tissues, of which 15.4% had occult involvement in the uterus. In patients with abnormal appeared uterine, 95.5% had pathological uterus involvement. Therefore, hysterectomy is recommended when the presence of abnormal appeared uterus and comprehensive consideration should be taken when uterus is normal. Evolvement of assisted reproductive technology enables the partial replacement of fallopian tube function. In present study, 71.3% of cases showed pathologic involvement of fallopian tubes and 68% had abnormal appearance, of which 94.2% had pathologic confirmed involvement. Surprisingly, these involvement was present in 22.8% of patients with bilateral normal appeared fallopian tubes. Thus, suggestions from fertility specialists might be included for fallopian tube resection.

This study had several limitations. First, the retrospective single-centered analysis with a limited sample size may lead to biases. Therefore, larger multicenter prospective studies are necessary to gain a better understanding of the impact of ovarian involvement. Second, since BSO was performed in all included patients, the actual impact of ovarian preservation in PMP was unknown. Therefore, comparison of the prognosis outcome in patients with normal appeared ovaries without oophorectomy should be performed. Third, this study did not explore the molecular events of PMP in detail. Considering the recent advancements in multi-omics technologies, gene profiling and cell-subtype comparisons of different grades PMP tissues could be beneficial in identifying novel disease markers, elucidating disease mechanisms, and discovering possible therapeutic targets.

## 5 Conclusions

In conclusion, our study confirmed that strong consideration should be given to removal of gynecologic organs, including uterus, fallopian tubes and ovaries, in cases of appendiceal peritoneal disease even when normal in appearance, after taking into account each individual patient’s fertility concerns.

## Data Availability

The original contributions presented in the study are included in the article/**Supplementary Material**. Further inquiries can be directed to the corresponding author.
