# Supplementary material for: Prophylactic oophorectomy is recommended in female patients with pseudomyxoma peritonei of an appendiceal origin
Source: Front Oncol. 2025 Jul 24;15:1485694. doi: 10.3389/fonc.2025.1485694 (PMC12329376; doi:10.3389/fonc.2025.1485694)
Supplement: Supplementary file 1 [file DataSheet1.docx]

Supplementary Table 2. Relationship between macroscopic and microscopic ovary involvement according to lateral position classification

| **Macroscopic** | Bilateral | | | Left | | | Right | | | |
| --- | --- | --- | --- | --- | --- | --- | --- | --- | --- | --- |
|  | 116(91.3%) | | | 5(4%) | | | 6(4.7%) | | | |
| **Microscopic** | Bilateral | Left | Right | Bilateral | Left | Right | Bilateral | Left | Right | No |
|  | 112(96.6%) | 1(0.9%) | 3(2.6%) | 2(40%) | 3(60%) | 0 | 0 | 0 | 5(83.3%) | 1(16.7%) |
