# Supplementary material for: Prophylactic oophorectomy is recommended in female patients with pseudomyxoma peritonei of an appendiceal origin
Source: Front Oncol. 2025 Jul 24;15:1485694. doi: 10.3389/fonc.2025.1485694 (PMC12329376; doi:10.3389/fonc.2025.1485694)
Supplement: Supplementary file 2 [file DataSheet2.docx]

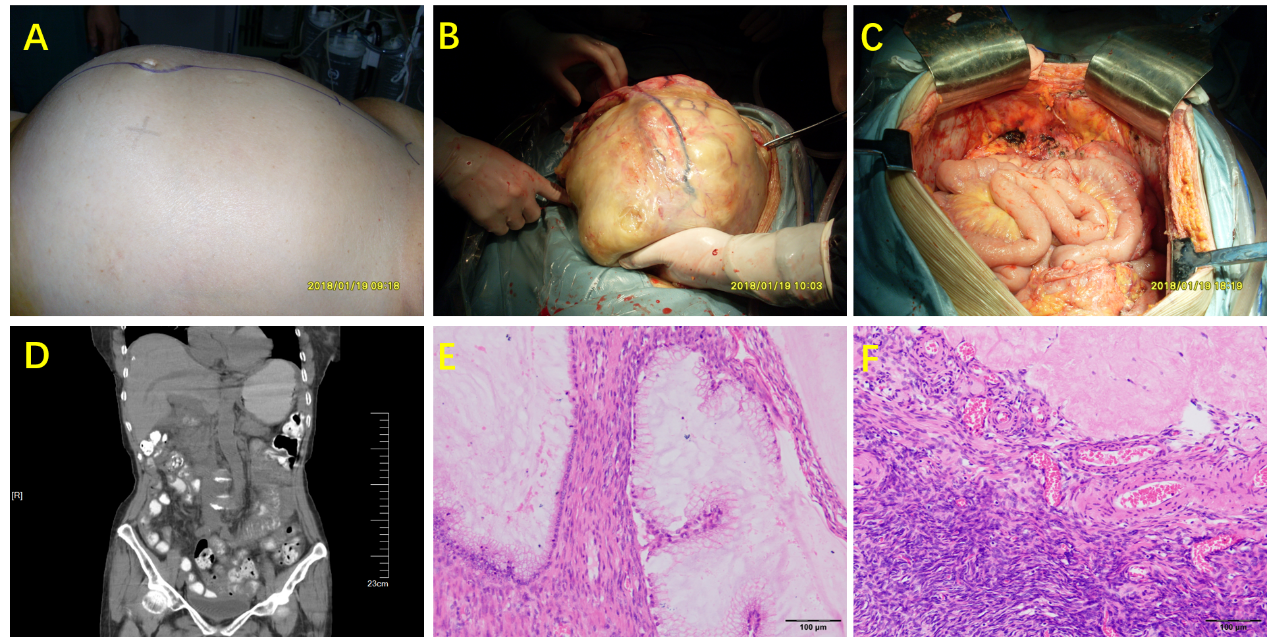


**Supplementary Figure.1** Surgical procedure (complete CRS surgery lasting for approximately 10 h) and pathological analysis of PMP of an appendiceal origin: (A) preoperative abdominal status; (B) ovarian involvement in the peritoneal cavity; (C) postoperative abdominal status; (D) abdominal and pelvic CT scan 6 months after cytoreduction therapy; (E) Hematoxylin & eosin (H&E) staining of the affected ovarian tissue specimens: low-grade cytologic atypia (200× magnification); (F) H&E staining of normal ovarian tissue specimens.
